# Pulpotomy versus pulpectomy in carious vital pulp exposure in primary incisors: a randomized controlled trial

**DOI:** 10.1186/s12903-024-04116-w

**Published:** 2024-03-20

**Authors:** Lamia Khairy Gadallah, Adel Elbardissy, Mohamed Abo Elyazeed, Ahmad Abd Alsamad, Mahmoud Hamdy

**Affiliations:** 1 Researcher of Pediatric Dentistry, Orthodontics and Pediatric Dentistry Department, National Research Centre, Elbuhouth st, Dokki, Cairo 12622 Egypt; 2 Pediatric Dentistry and Dental Public Health, Faculty of Dentistry, Cairo University, Dokki, Egypt; 3 Orthodontics and Pediatric Dentistry Department, National Research Centre, Dokki, Egypt; 4 Oral and Maxillofacial Radiology, Faculty of Dentistry, Cairo University, Dokki, Egypt; 5 Pediatric Dentistry and Dental Public Health Department, Faculty of Dentistry, Cairo University, Dokki, Egypt

**Keywords:** Primary incisors, Pulpotomy, Pulpectomy, Root canal therapy

## Abstract

**Background:**

Pulpotomy as a minimally invasive pulp therapy technique is the treatment of choice for carious pulp exposures, however many pediatric dentists perform pulpectomies in vital primary incisors. The aim of this split mouth randomized controlled study was to compare formocresol pulpotomy and zinc-oxide and eugenol pulpectomy in the treatment of vital pulp exposure in primary incisors.

**Methods:**

Contralateral pairs of incisors were randomly assigned to receive pulpotomy or pulpectomy in children aging from 18 to 66 months old and were followed up for 12 months.

**Results:**

39 pairs of incisors were included. Clinical and radiographical success rates showed no statistical significant difference (*p* = 1, *p* = 0.8 respectively). Relative risk measures for clinical success rates (RR = 1.03, 95%CI 0.87 to 1.23) and for radiographic success rates (RR = 1.03, 95%CI 0.83 to 1.29) with CIs including number one showing no difference between the two groups. The Survival rate using Kaplan-Meier survival analysis score showed 82% for pulpotomy and 74% for pulpectomy at 12 months (*P* = 0.2).

**Conclusions:**

Both pulpotomy and pulpectomy techniques can be used successfully in the treatment of carious vital pulp exposure in primary incisors.

**Trial registration:**

The trial was retrospectively registered in Clinicaltrials .gov with this identifier NCT05589025 on 21/10/2022.

## Introduction

Dental caries is a worldwide public health challenge, especially among young children. Early childhood caries (ECC) is a serious public health problem in both developing and developed countries [1]. Children’s quality of life can be seriously affected by such problem [2] .

Treatment of ECC can be achieved through different types of intervention. Various forms of pulp treatment were tried to treat or remove the pulp when caries extend to involve the pulp [3]. The deeper understanding of the dental pulp pathophysiology and its innate ability for healing when the insult is removed, together with the presence of bioactive materials, makes the concept of minimal invasive vital pulp therapy is now prevailing in the treatment of carious pulp exposures even with mature permanent teeth [4]. Besides that the American Academy of Pediatric Dentistry (AAPD) guidelines on pulp therapy states that pulpotomy is indicated for vital pulp therapy of primary teeth diagnosed with a normal pulp or reversible pulpitis [5].

Formocresol has been the popular material of choice for the pulpotomy technique. It was otherwise proved as “gold standard” in pediatric dentistry, may be because of its ease in use and excellent clinical success but this clinical success rate has always been in close observation due to its safety considerations and to the availability of the newer materials in the clinical market [3]. Some researches had showed that formaldehyde, the primary component in formocresol is probably not a potent human carcinogen under low exposure conditions and there is an inconsequential risk associated with formaldehyde use in pediatric pulp therapy [6, 7]. Moreover, according to the latest AAPD guidelines on vital pulp therapy in primary teeth, still formocresol and mineral trioxide aggregate (MTA) is the only two strongly recommended medicaments for use in pulpotomy [5]. Although MTA has excellent sealing ability together with its regenerative potential but there are some challenges concerning it as its long setting time, difficult handling characteristics, difficulty of removal once set, and high material cost [8]. Also, formocresol is widely used where 82% of graduate pediatric dental residency programs still utilize it for pulpotomy procedures in primary teeth [9].

Zinc-oxide and eugenol (ZOE) is the most commonly used root canal filling material for the pulpectomy procedure in primary teeth [10].

The claims that many pediatric dentists think pulpotomies don’t work in primary anterior teeth is not supported by high-quality evidence research [11]. These claims included that the remaining pulp tissues in a pulpotomized incisor may offer a source for acute inflammatory reaction. Additionally, there is no clear anatomical demarcation between the coronal and radicular compartments of the pulp in primary incisors, compared to primary molars, traumatic amputation of the coronal pulp, mechanical pressure on incompletely removed coronal pulp and poor diagnosis are all important causes for the clinical failure of pulpotomies [12, 13]. Moreover, many in the practicing community perform pulpectomies for the pulp treatment of carious vital primary anterior teeth [14].There are few studies that have compared pulpectomies with pulpotomies in vital primary incisors [15] .

The purpose of this study was to compare formocresol pulpotomy and zinc-oxide and eugenol pulpectomy in the treatment of vital pulp exposure in primary incisors clinically in terms of pain, pathological tooth mobility, soft tissue pathology and radiographically in terms of periapical radiolucency and pathological root resorption. We hypothesized that formocresol pulpotomy would be as effective as zinc-oxide and eugenol pulpectomy in the treatment of vital pulp exposure in primary incisors.

## Methods

### Trial design

This is a randomized controlled trial with split mouth design.

This trial was conducted in the Pediatric Dentistry and Dental Public Health Department. The trial was registered in Clinicaltrials .gov with this identifier NCT05589025 on 21/10/2022.The study was approved by the research ethics committee of the National Research Centre, Egypt with a registration number 14154 on 1/11/2014.

### Inclusion criteria

Patients’ eligibility criteria were medically free patients, aging from 18 to 66 months old, with two or more carious vital primary maxillary incisors where exposure of the vital pulp following the removal of dental caries was inevitable. They were selected from the outpatient clinic and from the patients referred to be treated under General anesthesia.

Included incisors had no history of spontaneous pain, lingering provoked pain, pain on percussion, fistula or sinus tract, no history of trauma, no periapical radiolucency, pathologic root resorption or pulp calcification, no signs of inflammation extending beyond the coronal pulp, physiologic resorption does not exceed one third of the root and teeth were restorable with crowns.

### Sample size

Sample size was calculated using PS Computer Program [16].A study of matched cases and controls was planned. Prior data indicated that success rates among controls were 0.78 [11, 17]. If the true success rate for experimental subjects is 1, then we needed to study 31 pairs to be able to reject the null hypothesis that the success rates for experimental and control subjects are equal with probability (power) 0.8. The Type I error probability associated with this test of this null hypothesis is 0.05 McNemar’s chi-squared statistic was used to evaluate this null hypothesis. This number has been increased to a total sample size 39 in each group, to allow for losses of around 25%.

The procedure, possible discomforts or risks, as well as possible benefits were explained completely to the parents or legal guardians. An informed consent was obtained from the parents or legal guardians before participation in the study.

The child participants and legal guardian of each participating child were blinded to the type of treatment they received while it was not possible for the operator or the radiographic assessors to be blinded due to the nature of the treatment received.

### Randomization

An incisor in each pair was randomly assigned by a coin toss to either the intervention (pulpotomy group) on the head side or the control (pulpectomy group) on the tail side with the contralateral paired incisor being designated to the other treatment group. The tossing was performed by the assistant before the access cavity preparation.

### Procedure

Clinical examination and preoperative periapical radiographs were performed for eligible patients. After induction of anesthesia, teeth were properly isolated with cotton rolls and suction as rubber dam may negatively affect their behavior and increase the level of dental anxiety in young children [18]. Complete removal of caries or undermined enamel was performed before access cavity preparation. For the pulpotomy group, the pulpotomy procedure performed was a modification of that described by Pinkham et al. [11] where pulp chamber was unroofed using a no. 330 sterile bur in a water-cooled high-speed handpiece. The entire roof of pulp chamber and overhanging dentinal remnants over the pulp horns were removed. After the completion of the access cavity, coronal pulp was extirpated using a sharp excavator. Any residual coronal pulpal tissue was removed using a sterile round bur in a slow-speed handpiece to a depth of few millimeters below the free gingival margin. Hemostasis was achieved with a water-dampened cotton pellet. If hemostasis was not achieved after the initial application of the cotton pellet, the case was eliminated from the study. Following hemostasis, a cotton pellet, moistened with formocresol (formaldehyde 37%, cresol ˂50%, glycerin and isopropyl alcohol; PREVEST DenPro, India) was applied for 3 min and removed. A zinc- oxide and eugenol (ZOE) base (Caryosan zinc oxide-eugenol cement; Spofadental, The Czech Republic) was placed over the amputation site. Thereafter, a glass ionomer base (Glass ionomer cement, Ningbo Gaoju, China) was applied. For the pulpectomy group, the pulpectomy procedure used herein was a modification of that described by Payne et al. [13]. Pulp chamber was unroofed using a no. 330 sterile bur in a water-cooled high-speed handpiece. The entire roof of pulp chamber and overhanging dentinal remnants over the pulp horns were removed. An initial endodontic K-file (MANI®, Japan) fitting snugly in the canal was introduced inside it. In most cases, the pulp tissue was removed completely on the first attempt. If the first attempt was unsuccessful, the procedure was repeated, and canals were generally enlarged three sizes past the initial file to eliminate the organic remnants. Copious irrigation with a light flow of sterile 0.9% NaCl-solution was used throughout the procedure. At the end, the canals were dried using paper points. The canals were filled with ZOE paste (Caryosan zinc oxide-eugenol cement; Spofadental, The Czech Republic) and it was delivered with a Lentulo spiral paste filler (MANI paste carrier, MANI INC, Japan) where it is inserted in the canal to a point just short of the apex. A glass ionomer base (Glass ionomer cement, Ningbo Gaoju, China) was applied. All teeth were immediately restored with preveneered stainless steel crowns (Nu Smile crowns, USA). All crowns were cemented with glass ionomer cement (GC Fuji IX Capsule, GC, Japan).

### Outcomes

The primary outcome for this study was a core set of component outcomes [19]. Clinically including pain, pathological tooth mobility and soft tissue pathology (gingival swelling, sinus, fistulous tract), moreover radiographically including periapical radiolucency and pathological root resorption. The secondary outcomes included tooth survival in both groups and pulp canal obliteration in pulpotomy group.

For follow up: Clinical evaluation was performed on all primary incisors during the follow-up visits at one, six and twelve months post-operatively while radiographic evaluation was performed at six and twelve months follow up visits.

For radiographic evaluation, the radiographs were taken with a size 0 or 1 periapical films (D-speed Film, Ultra-speed Carestream Dental, USA) using the bisecting angle technique. The radiographs were scanned on a viewer and transmitted to a computer hardware to be properly saved and to be kept as a record until the completion of the trial in case of any distortion of the radiographs from the long storage. The evaluation was performed by two independent assessors and differences were solved by consensus. Data analysis was performed on the consensus scores.

For Statistical analysis, Chi square test was used to compare between the two groups. The significance level was set at *P* ≤ 0.05. Also estimated effect size was calculated with 95% Confidence Interval. Kaplan–Meier used for survival analysis. Statistical analysis was performed with IBM® SPSS® (SPSS Inc., IBM Corporation, NY, USA) Statistics Version 25 for Windows.

## Results

The sample size of this study was 39 pairs of incisors in 31 patients (20 males and 11 females) aging from 18 to 66 months with a mean age of 48.9 ± 13.8 months old. 10 patients were treated under general anesthesia due to their very young age and 21 patients were treated under local anesthesia.

From the patients initially assessed for eligibility, some patients were excluded from the study by the operator for reasons including no pulp exposure following caries removal (three cases) and pulp necrosis (one case). No parents declined when offered participation in the study.

The 39 pairs of incisors were randomized where 39 incisors received pulpotomy and 39 incisors received pulpectomy including 50 central incisors (25 pairs) and 28 lateral incisors (14 pairs). The patients were treated by two pediatric dentists.

At the 6 months follow up period, six pairs of incisors missed their follow up, this left 33 pairs available for assessment at six months. While at 12 months, two patients who missed there six month follow up showed up, only one patient who received his six months follow up did not return for his 12 months follow up, meanwhile two patients were excluded at 12 months follow up as they received trauma to the treated incisors. This left 32 pairs of incisors available for assessment at 12 months follow up period.

**Fig. 1:**
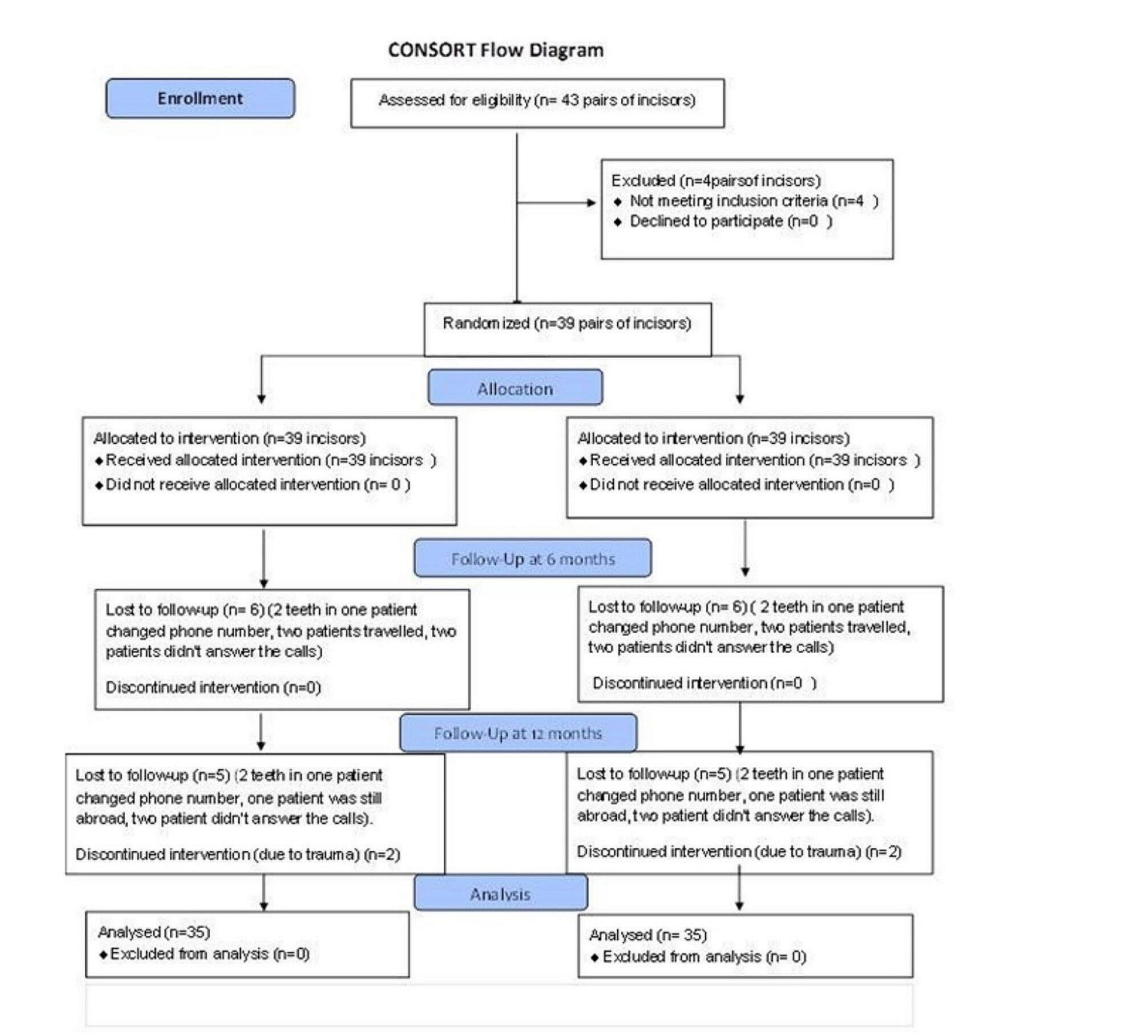
Consort flow diagram

The dropped-out patients were patients with 4 pairs of incisors that missed their 6 and 12 months follow ups. Data about these 4 pairs of incisors were missing, this left us 35 pairs of incisors included in the analysis as shown in Fig. 1: CONSORT Flow Diagram.

Clinical evaluation was performed by one pediatric dentist which was blinded to the treatment received. In the clinical evaluation, pain and soft tissue pathology was not reported in relation to any incisor in both groups while pathologic mobility was the only negative finding that occurred as shown in Table 2. The P value showed no statistical significant difference in clinical success between pulpotomy and pulpectomy groups as shown in Table 1. Relative risk measure (RR) for clinical success at 12 months equals 1.03 95% CI 0.87 to 1.23.

**Table 1:** Frequency (N) and percentage (%) for clinical and radiographic success rates for pulpotomy and pulpectomy groups

			Groups				p-value
			Pulpotomy		Pulpectomy		
			N	%	N	%	
Clinical success	6 Months	Yes	32	97.0%	29	87.9%	0.3990 NS
		No	1	3.0%	4	12.1%	
	12 Months	Yes	29	90.6%	28	87.5%	1.00 NS
		No	3	9.4%	4	12.5%	
Radiographic success	6 Months	Yes	31	93.9%	27	81.8%	0.2383 NS
		No	2	6.1%	6	18.2%	
	12 Months	Yes	27	84.4%	26	81.2%	0.8150 NS
		No	5	15.6%	6	18.8%	

*= Significant, NS = Non-significant

**Fig. 2:**
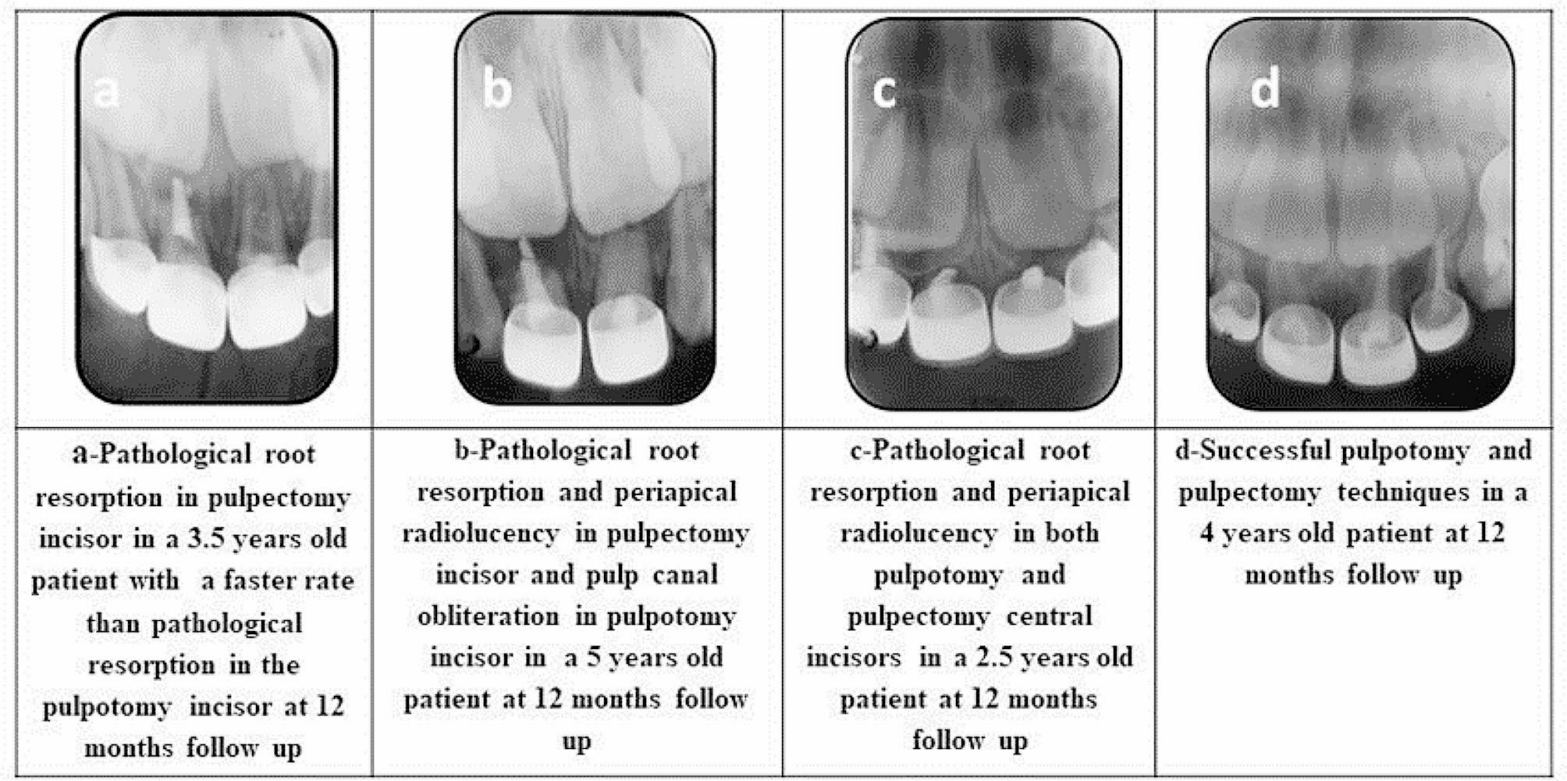
Radiographic findings in pulpotomy and pulpectomy incisors

For radiographic evaluation, pathologic external root resorption and periapical radiolucency were reported in both groups while no incisors were reported with internal resorption in the pulpotomy group and only one incisor showed pulp canal obliteration in the pulpotomy group as shown in Table 2; Fig. 2. The P value showed no statistical significant difference in radiographic success between pulpotomy and pulpectomy groups as shown in Table 1. Relative risk measure (RR) for radiographic success at 12 months equals 1.03 95% CI 0.8305 to 1.29.

**Table 2:** Frequency (N) and percentage (%) for clinical and radiographic outcomes for pulpotomy and pulpectomy groups

			Groups				p-value
			Pulpotomy		Pulpectomy		
			N	%	N	%	
Pain	6 Months	No	33	100.0%	33	100.0%	1.00 NS
		Yes	0	0.0%	0	0.0%	
	12 Months	No	32	100.0%	32	100.0%	1.00 NS
		Yes	0	0.0%	0	0.0%	
Soft tissue pathology	6 Months	No	33	100.0%	33	100.0%	1.00 NS
		Yes	0	0.0%	0	0.0%	
	12 Months	No	32	100.0%	32	100.0%	1.00 NS
		Yes	0	0.0%	0	0.0%	
Pathologic mobility	6 Months	No	32	97.0%	29	87.9%	0.3990 NS
		Yes	1	3.0%	4	12.1%	
	12 Months	No	29	90.6%	28	87.5%	1.00 NS
		Yes	3	9.4%	4	12.5%	
Pathological external resorption	6 Months	No	31	93.9%	27	81.8%	0.2383 NS
		Yes	2	6.1%	6	18.2%	
	12 Months	No	27	84.4%	26	81.2%	0.8150 NS
		Yes	5	15.6%	6	18.8%	
Periapical radiolucency	6 Months	No	31	93.9%	27	81.8%	0.2383 NS
		Yes	2	6.1%	6	18.2%	
	12 Months	No	27	84.4%	26	81.2%	0.8150 NS
		Yes	5	15.6%	6	18.8%	
Pulp canal obliteration	6 Months	No	32	96.9%	NA	NA	-
		Yes	1	3.03%	NA	NA	
	12 Months	No	31	96.8%	NA	NA	-
		Yes	1	3.1%	NA	NA	
Internal resorption	6 Months	No	33	100.0%	NA	NA	-
		Yes	0	0.0%	NA	NA	
	12 Months	No	32	100.0%	NA	NA	-
		Yes	0	0.0%	NA	NA	

The relative risk for overall success (Clinical and Radiographical) at12 months equals 1.03 95% CI 0.8305 to 1.29. The CI included 1 in all outcomes then the risk of success is equal in both pulpotomy and pulpectomy and there is no difference between the two groups.

Kappa used for measurement of agreement showed good agreement between the two radiographic assessors at kappa = 0.788 prior to reaching a consensus score.

**Fig. 3:**
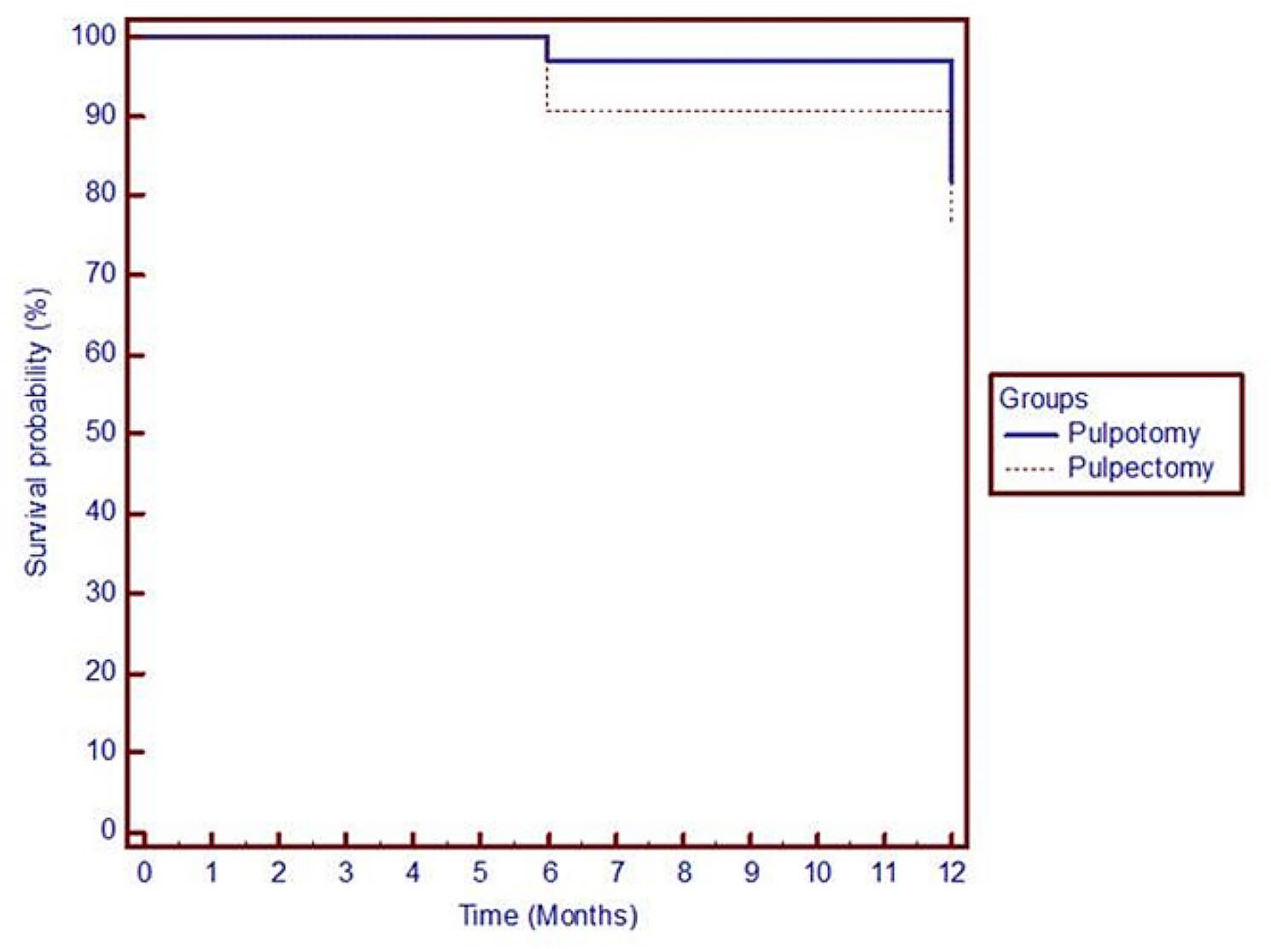
Kaplan-Meier survival analysis

The Survival rates using Kaplan-Meier survival analysis showed no statistical significant difference between pulpotomy and pulpectomy groups as shown in the Fig. 3, where survival rates for pulpotomy at 12 months was 82% versus 74% for pulpectomy.

Subgroup analysis for central and lateral incisors was performed and showed that no failure rates were reported for lateral incisors at 6 or 12 months follow up periods.

## Discussion

Pulpotomy and root canal therapy have been both performed as techniques for the management of asymptomatic vital primary incisors with large carious lesions where removal of caries will lead to pulp exposure [13].

The aim of this study was to compare between formocresol pulpotomy and zinc oxide and eugenol pulpectomy clinically and radiographically in the treatment of carious vital pulp exposure in primary incisors. This study showed that there is no statistical difference in clinical and radiographical success rates of both groups.

The preference of many pediatric dentists to perform pulpectomy in primary incisors was due to that they were taught to do so in their pediatric dentistry residencies and not due to evidence from high quality research [11].

Up till now, there are only four randomized controlled trials that have compared pulpotomy and pulpectomy outcomes in vital primary incisors [11, 13, 14, 17].

In this study, the clinical success rates were similar to previous studies where there was no statistical significant difference between the two groups [11, 13, 14, 17].

It worth noting that in our study out of the four teeth (12.5%) that failed in pulpectomy group, three of them was corresponding to the other three contralateral teeth (9.4%) that have failed in pulpotomy group. It was also shown in Howley et al. study in 2012 [11] as their investigation was a split mouth design and found that in two patients, both treatments failed illustrating the importance of having teeth in each treatment group represented in each subject.

For radiographic assessment, this study showed no statistically significant difference in radiographic success rates between the two groups although there were higher numbers of successful radiographic outcomes for formocresol pulpotomy than pulpectomy at each observation interval. These results were in agreement with the results of Nguyen et al. study [14] in 2017 with an 18 months follow up and Howley et al. study [11] in 2012 with a 23 months follow up.

While according to Aminabadi et al. study [13] in 2008, radiographic success rates were higher in pulpectomy group than in pulpotomy group with statistical significant difference at two year follow-up. Also Casas et al. study [17] in 2004 showed higher radiographic success in pulpectomy group than in pulpotomy group but not statistically different at 2 years follow up.

The difference in the success rates between our study and the study of Aminabadi et al [13] and Casas et al. [17] was firstly suggested to be due to the difference in the final restoration used as it is an acid etch resin restoration in those studies.

These resin restorations could significantly impact the biological seal and treatment success rates. Shrinkage associated with resin restorations may lead to the pulp-treated teeth being more susceptible to marginal leakage where bacterial toxins can permeate through faulty restorations and the intermediate restorative material layer and affect the radicular pulp therefore microleakage would increase the susceptibility for failure in pulpotomy treated teeth vs. those treated with pulpectomies [11].

Although this suggestion is scientifically acceptable but it was refuted by the results of Nguyen et al. study [14] where acid etch resin restoration was their final restoration and the success rates were high in both groups.

The difference in the success rates between this study and the study of Casas et al. [17] could be attributed also to the very small final sample size. The 23% difference in radiographic outcomes for both treatments was equivalent to only 2 teeth. The 36% drop out in the pulpotomy group and 48% drop out in the root canal treatment group at 2 years follow up imposed high risk of bias to their study.

Pathological tooth mobility was the only negative clinical finding in our study, and it was related to teeth with extensive pathological resorption. All teeth exhibiting pathological external resorption were accompanied with periapical radiolucency. No teeth were rated with questionable periapical radiolucency, and radiographic assessors of this study highlighted that subtle pathologic changes as minute radiolucenies in the incisal region couldn’t be identified due to close proximity of the developing tooth bud and its follicle and superimposition of these anatomical structures. Radiographic assessors considered external root resorption to be pathological according to the age of the patient, comparison with the contralateral tooth and presence of any another pathological sign as frank periapical radiolucency.

In this study pathological root resorption was observed to be at faster rate in the pulpectomized incisors rather than pulpotomized ones. While the rate of resorption of zinc oxide and eugenol in some pulpectomized incisors was slower than root resorption.

Retention of ZOE was reported in many studies [20–22]. All the incisors in this study were filled to the apex or slightly underfilled. Extent of ZOE was stated to affect the prognosis of pulpectomy. Overfilling of the zinc oxide-eugenol paste and its extrusion beyond the root apex was reported to cause pulpectomy failure. Filling of the canals to the apex is the best while underfilling is better than overfilling due to the probability of extrusion of the ZOE beyond the root apices and causing irritation [12, 23].

Moreover, this study showed that all the clinical and radiographical failures that occurred were related to central incisors. The higher failure rates that are related to central incisors in relation to lateral incisors were previously reported [24]. There are two factors suggested to be contributing for the poor prognosis of pulp therapy in central incisors. The physiologic process of root resorption that occurs early in central incisors rather than lateral incisors and the presence of inflammatory microenvironment that could play a role in the differentiation of odontoclasts together with the position of the central incisor in the dental arch and the mechanical forces applied on it [25].

Pulpotomy technique may reduce treatment time when compared to the pulpectomy technique in primary incisor [14].

As for the cost, the American Dental Association’s Survey of Dental Fees 2009 showed that the mean cost of primary anterior pulpectomies is 32% greater than for pulpotomies [11].

Taking into account the success rates, reduced time and cost of pulpotomy, choosing of pulpotomy instead of pulpectomy for the treatment of vital pulp exposure in primary incisors seems justified.

Limitations of this study design included no blinding of operators providing treatment or raters assessing radiographic outcomes. However, blinding was not possible, as the treatment techniques are easily distinguishable when they are performed and when treated teeth are assessed radiographically .Also, difficulty of radiographic interpretation of primary teeth in the incisal region with a two-dimensional radiograph was another limitation. A three-dimensional imaging techniques as computerized tomography would be the best for proper assessment but taking into consideration its high cost and the higher radiation dose, it was considered an unnecessary additional hazard for the patients.

Further studies comparing between pulpotomy and pulpectomy with a longer follow up period till exfoliation time are recommended.

## Conclusions

There was no significant difference in the success rates of pulpotomies and pulpectomies in the pulp treatment of carious vital pulp exposure in primary incisors. Formocresol pulpotomy is an alternative treatment to pulpectomy in vital primary incisors.

## Data Availability

The datasets used and/or analyzed during the current study are available from the corresponding author on reasonable request.
